# Valproic Acid Ameliorates Locomotor Function in the Rat Model of Contusion via Alteration of *Mst1*, *Bcl-2*, and *Nrf2* Gene Expression

**DOI:** 10.52547/ibj.25.4.303

**Published:** 2021-02-28

**Authors:** Ali Mardi, Alireza Biglari, Reza Nejatbakhsh, Alireza Abdanipour

**Affiliations:** 1 Department of Molecular Medicine and Genetics, School of Medicine, Zanjan University of Medical Sciences (ZUMS), Zanjan, Iran;; 2 Department of Anatomy, School of Medicine, Zanjan University of Medical Sciences (ZUMS), Zanjan, Iran

**Keywords:** Bcl-2, Contusion, Mst1, Nrf2, Valproic acid

## Abstract

**Background:**

In animal models of inflammatory diseases, *Mst1* facilitates the programmed cell death as a novel pro-apoptotic kinase. This research aimed to determine the expression level of *Mst1* gene in a rat model of SCI treated with VPA.

**Methods:**

Severe rat model contusion was used for evaluation of the neuroprotective effect of valproic acid. The BBB test, was performed to determine locomotor functions. H&E staining and TUNEL assay were performed to detect cavity formation and apoptosis, respectively. The mRNA levels of the genes *Mst1*, *Nrf2*, and *Bcl-2* were evaluated, using quantitative RT-PCR.

**Results:**

The results revealed that *Mst1* gene expression and TUNEL-positive cells in the VPA-treated group were significantly reduced as compared to the untreated group (*p* ≤ 0.05).

**Conclusion:**

Our findings indicate that VPA has therapeutic potential and can be a candidate for the treatment of neurodegenerative disorders and traumatic injury as a promising drug.

## INTRODUCTION

Evidence has shown that tissue damage and cell death through necrosis and apoptosis continue in the second phase of SCI. However, apoptosis begins in the early hours after injury and continues for weeks, and caspase-3 activation is one of the essential factors in apoptosis. *Mst1* is a key pro-apoptotic gene stimulating caspase-3 activation^[^^1^^]^. In addition, the inhibition of the upper and lower cascading events of caspase-3 can be used as a therapeutic target in treating SCI^[^^2^^]^. Using anti-inflammatory agents such as VPA, as an inhibitor of histone deacetylase, can hinder apoptosis^[^^3^^]^. Mst1 is a serine/threonine protein kinase participates in apoptotic induction. It enters the nucleus after a caspase-mediated cleavage and induces condensation of chromatin, followed by fragmentation of DNA^[^^1^^]^. Mst1 protein has been reported to be able to induce the mitochondrial-dependent apoptosis pathway through caspase-dependent and independent pathways. The *Nrf2* gene influences cell proliferation, cell growth, and cell metabolism control via the phosphatidylinositol-3-kinase/ protein kinase pathway and increases the expression of the anti-apoptotic protein Bcl-2^[^^4^^]^.

Acute SCI is resulted from secondary progressive tissue destruction and caused by inflammatory response. Therefore, to reduce secondary tissue damage, a logical approach is to restrict this response. Ischemia-related inflammation induces tissue and cellular edema, oxidative damage, and apoptosis depending on the size of the lesion. One strategy to reduce neuronal cell death (apoptosis/necrosis) is the use of biomolecules or protective agents that reduce the function of pro-apoptotic genes. 

Herein, we decided to investigate the effect of VPA on Mst1 expression in a rat model of SCI due to the efficacy of VPA in enhancing motor function and decreasing the cell death after SCI, The important role of *Mst1* in apoptosis and neuronal damage has also been explored in this study. 

## MATERIALS AND METHODS

**Contusion model**

Wistar female rats (n = 32, weighing 200-250 g, aged 8-12 weeks old) were assigned to four experimental groups (n = 8 per group): Severe contused animals without any treatment (untreated), laminectomy (control), contused animals treated with normal saline (0.5 ml; vehicle) and contused animals treated with VPA (400 mg/kg; Sigma, USA). All injections were given intraperitoneally with a final volume of 0.5 ml and continued once daily for seven days, starting three hours after the procedure. The optimal dose of VPA was chosen in accordance with a previous study^[^^5^^]^. Sever contusion SCI model was applied using previously mentioned weight-drop procedure^[^^6^^,^^7^^]^. The BBB open-field test was used to evaluate locomotor activity between experimental groups for four minutes. Rats videotaped with a digital video camera (Canon EOS 80D; japan) at 3, 7, 14, 21, and 28 days post SCI. Repeated tests of variance analysis (ANOVA) accompanied by Tukey's post-hoc test reported the significant differences in scores. 

**Histological assessments**

Rats were anesthetized 28 days after SCI and then transcardially perfused with heparin-containing saline solution (1 unit/ml), followed by a fixative solution (4% paraformaldehyde). The lesion area was removed and post fixed in a same solution for next 12 hours. Tissues passed through an automated processor (Leica TP 1020, Germany) and were embedded in paraffin blocks. Serial sections of spinal cords were prepared and then dewaxed with chloroform for cavity evaluation and stained with H&E. The percentage volume of the cavity in the 3000-μm length of the spinal cord (a total of 30 sections, including the rostral, central and caudal regions) was evaluated using the software Image J and the Cavalieri method (equation 1; V_sp_, volume spheroid; a, measured area; d, intersection distance)^[^^8^^]^. 

 V_sp_ = a × d.           (Eq. 1)

The TUNEL assay was conducted according to the manufacturer's protocol (Roche, Germany). Using an Olympus BX61 fluorescence microscope, five non-overlapping fields around the injury site were randomly chosen. 

**Quantitative RT-PCR**

In all the groups, 1,000 ng of purified RNA (DNA free) was applied to synthesize 20 µl of cDNA, using a cDNA synthesis kit (Fermentas, USA). All quantitative PCR reactions were performed in triplicate, and the final volume for each reaction was 12.5 µl, containing 6 µl of RealQ Plus 2× Master Mix Green (Ampliqon; Denmark), 0.76 µl of each forward and reverse primer (0.3 µM; Table 1), 4.5 µl of RNase-free water, and 0.5 µl of cDNA (final concentration of 25 ng per quantitative PCR). The PCR reaction (Applied Biosystems 7500, USA) was conducted for 40 cycles. We used the Pfaffl method (equation 2) for evaluating relative changes in mRNA levels^[^^9^^]^. *Mst1*, *Nrf2*, and *Bcl-2* mRNA were normalized against GAPDH, and the samples of laminectomy SCI group were employed as a calibrator. 

$\mathrm{Ratio}=2^{-\left(∆∆C_{t}\right).∆∆C_{t}={∆C}_{t_{\mathrm{reference}}}-∆C_{t_{\mathrm{target}}}}$           (Eq.2)

**Statistical analysis**

Using SPSS15 software, the statistical analysis was carried out. All data were presented as mean ± SEM. A one-way ANOVA accompanied by Tukey's post hoc comparison and student's *t*-test analysis was employed to compare different means in the groups. Values of *p* ≤ 0.05 were considered as statistically significant.

**Ethical statement**

All the experimental procedures were carried out in compliance with Zanjan University's (ZUMS) ethical guidelines (ethical code: A-12-973-5).

**Table 1:** Primer sequences and PCR parameters

**Gene**	**Gene Accession no.**	**Sense 5 → 3**	**Anti-sense 5 → 3**
*Mst1*	NM_001107800.1	GCTAAAGTGAAGTGGACGGATACC	GGAACAGTTGCTACCAGAGTGTCAG
*Nrf2*	NM_031789.2	CACCAGTGGATCTGTCAGCTACTC	GTGGTGAAGACTGAGCTCTCAACG
*Bcl-2*	NM_016993.1	GTGGCCTTCTTTGAGTTCGGTG	ATCCCAGCCTCCGTTATCCTG
*GAPDH*	NG_028301.2	AACCCATCACCATCTTCCAG	GTGGTTCACACCCATCACAA

## RESULTS

**BBB score**

Recorded BBB scores showed a significant increase at 21 and 28 days post SCI (10.75 ± 1.06 and 9.37 ± 1.08, respectively) in the VPA-treated group compared to the contusion group. The numerical difference (delta number) between days 3 and 28 post SCI were significantly different between VPA-treated (10.68 ± 1.1) and contusion (1.58 ± 0.49) groups. 

**Histological assessment**

**Fig. 1:**
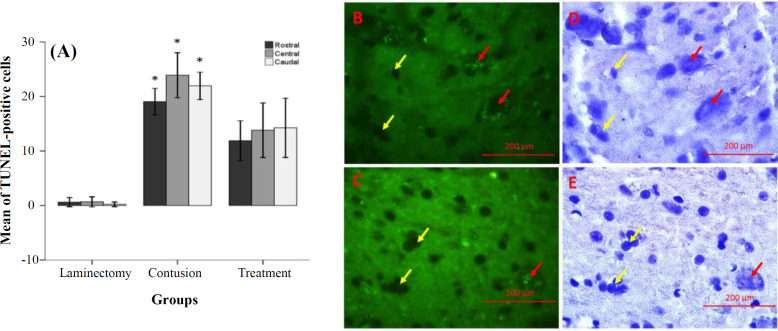
Effects of VPA on the inhibition of apoptosis 28 days post SCI (TUNEL assay, fluorescence microscope). (A) Bar graphs indicate the mean percentage of apoptotic cells in the experimental groups. Apoptotic cells were calculated in three (rostral, central and caudal) regions; (B and C) representative images of TUNEL staining (B, contusion and C, VPA); (D and E) counterstaining of the same field with hematoxylin. Red and yellow arrows show positive and negative cells, respectively. The bars indicate the mean ± SEM (^*^p ≤ 0.05 vs. VPA group; magnification 400×).

Analysis of cavity percentage revealed a significant difference between VPA-treated (5.35 ± 0.3) and contusion (19.26 ± 2.04) groups. A small population of apoptotic cells was observed in the laminectomy group (rostral: 0.61 ± 0.41, central: 0.65 ± 0.45, and caudal: 0.21±0.21); however, a large number of apoptotic cells were observed in the contusion group (rostra: 19.05 ± 1.21, central: 23.86 ± 2.05, and caudal: 21.95 ± 1.25), as demonstrated by TUNEL assay. The number of apoptotic cells significantly decreased in the VPA-treated group (rostral: 11.88±1.82, central: 13.8±2.45, and caudal: 14.24±2.71) compared to the contusion group. These results are demonstrated in Figure 1.

**Gene expression**

**Fig. 2:**
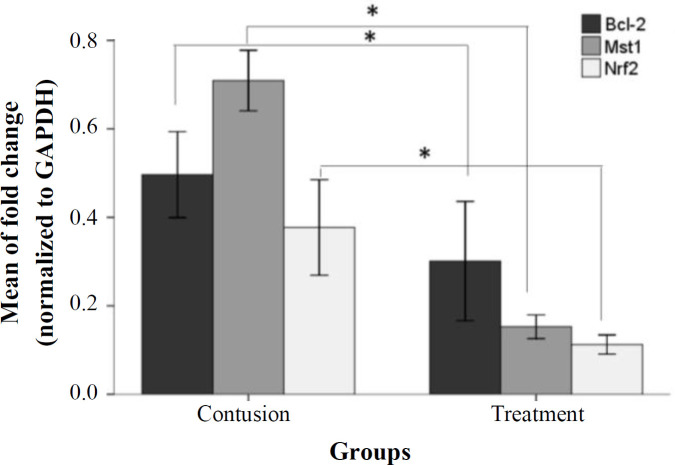
Quantitative RT-PCR results relative to the laminectomy group. The mRNA level of *Mst1*, *Nrf2*, and *Bcl-2* is presented as the relative expression normalized to GAPDH mRNA amplification. The bars indicate the mean ± SEM (^*^p ≤ 0.05 vs. VPA group).

Using quantitative RT-PCR, improvements in the expression of *Mst1*, *Nrf2*, and *Bcl-2* mRNA levels inall experimental groups were analyzed. Data relating to the laminectomy community were presented. In the VPA-treated group, *Mst1*, *Nrf2*, and *Bcl-2* mRNA expression (0.15 ± 0.01, 0.11 ± 0.01, and 0.3 ± 0.06, respectively) significantly decreased compared to the control group (0.7 ± 0.03, 0.37 ± 0.03, and 0.49 ± 0.04, respectively), as depicted in Figure 2 (*p* ≤ 0.05).

## DISCUSSION

In the chronic phase of SCI, cell death continues due to apoptosis. In this study, we have shown that VPA reduced *Mst1* gene expression and apoptosis rate after SCI. There are extensive findings regarding the activation of the mechanisms of apoptosis following SCI^[^^10^^]^. *Mst1* plays an important role in mediating apoptosis, but its precise role has not been well known^[^^1^^]^. Studies have indicated that apoptosis happens in the models of SCI and is followed by caspase-3 activation^[^^11^^,^^12^^]^. Lee *et al.*^[^^13^^] ^have suggested that VPA prevents the cell death and caspase 3 activation, reduces spinal cord lesions and improves locomotor function after SCI. For the advancement of novel approaches for the prevention and treatment of SCI, discovering pathways that can inhibit the progression of inflammation and apoptosis is important. MST1 is regarded as one of the proteins directly and indirectly associated with caspase-3^[^^13^^]^. 

Results from this study indicated that SCI can activate apoptosis in the lesion site and surrounding regions. *Mst1* apparently activates downstream targets such as JNK/p38, histone H2B, and FOXO^[^^14^^]^. *Mst1 *also induces apoptosis in cardiac myocytes by phosphorylation and *Bcl-xLL* inhibition^[^^15^^]^. Another group of regulatory proteins, such as anti-apoptotic *Bcl-2*, *Bcl-XL* and *Nrf2*, can protect apoptotic cell death. Several apoptotic signals converge on caspase activation, and this pathway is regulated by *Bcl-2*, *Bcl-XL* and *Nrf2*, as well^[^^16^^]^. Regarding the role of apoptosis in SCI, new therapies, i.e. the inhibition of genes involved in apoptosis signaling pathway such as *Mst1*, could result in the reduced cell death^[^^17^^]^. 

VPA is an anticonvulsant and a mood-stabilizing drug with proven neuroprotective and anti-apoptotic effects in rat SCI model and other neurological diseases^[^^18^^]^. In this study, we have displayed that VPA significantly reduces the expression of *Mst1* and subsequently decreases apoptosis compared to the untreated contused group. VPA is recognized as a strong histone deacetylase inhibitor^[^^3^^]^. Histone acetylation is a key mechanism for modification of chromatin structure and genes expression^[^^19^^]^. In our previous research, we have demonstrated that VPA would decreases the production of secondary damage in rat spinal cord trauma dependently on the dosage, resulting in improved locomotor score and recovery time^[^^20^^]^. To date, very limited study has been carried out on the function of VPA in the expression of the *Mst1* gene. Lee *et al.*^[^^21^^]^ have found that in a rat model SCI, 300 mg/kg of VPA increases the expression levels of Bcl-2 and Bax mRNAs. In the current study, VPA treatment was performed in the acute phase of injury, but apoptosis rate and gene expressions participating in apoptosis were evaluated in the chronic phase. 

*In vivo* documents have demonstrated that the lack of the *Mst1* increases spinal motor neuron survival after trauma, locomotor scores, and synapse survival^[^^22^^]^. MST1 proteins are mainly located within the cytoplasm, but during stress, they can be cleaved by caspase-3 and relocated into the nucleus^[^^23^^].^ One of the key factors involved in the inhibition of apoptosis proteins is *Nrf2*. This transcription factor, along with the increased expression of enzymes, is related to antioxidant and detoxification, prevents cell death and is considered as an anti-apoptotic factor^[^^24^^]^. Evidence has revealed that in the cytoplasm, Keap1 preserves *Nrf2*^[^^25^^]^. Our results indicated that the expression of *Nrf2* reduced in the valproic acid-treated group. This reduction may be due to decreased inflammation and apoptosis in the lesion areas. We can conclude that with the injection of valproic acid, the amount of cellular stress declines, resulting in the reduced expression of *Nrf2* and *Bcl-2* genes. 

In conclusion, VPA may be seen as a potential drug candidate for the treatment of neurodegenerative conditions. Moreover, pharmacological inhibition of *Mst1* can be used as a form of therapy for neurodegenerative disorders.
